# Supplementary material for: Multi‐dataset identification of innovative feature genes and molecular mechanisms in keratoconus
Source: J Cell Mol Med. 2024 Sep 19;28(18):e70079. doi: 10.1111/jcmm.70079 (PMC11412914; doi:10.1111/jcmm.70079)
Supplement: Supplementary file 2 — Material S2. [file JCMM-28-e70079-s002.zip › 7.GSEA analysis/5.RNF217-AS1/KEGG_NOTCH_SIGNALING_PATHWAY.html]

Details for gene set KEGG\_NOTCH\_SIGNALING\_PATHWAY[GSEA]

|  || Dataset | RNF217-AS1.group.cls#h\_versus\_l |
| Phenotype | group.cls#h\_versus\_l |
| Upregulated in class | h |
| GeneSet | KEGG\_NOTCH\_SIGNALING\_PATHWAY |
| Enrichment Score (ES) | 0.19093746 |
| Normalized Enrichment Score (NES) | 0.721084 |
| Nominal p-value | 0.7797619 |
| FDR q-value | 1.0 |
| FWER p-Value | 1.0 |
Table: GSEA Results Summary

  

Fig 1: Enrichment plot: KEGG\_NOTCH\_SIGNALING\_PATHWAY      
 Profile of the Running ES Score & Positions of GeneSet Members on the Rank Ordered List

  

| SYMBOL | TITLE | RANK IN GENE LIST | RANK METRIC SCORE | RUNNING ES | CORE ENRICHMENT || 1 | MFNG | na | 378 | 0.565 | 0.1195 | Yes |
| 2 | DTX1 | na | 1227 | 0.416 | 0.1909 | Yes |
| 3 | DLL4 | na | 6569 | 0.166 | 0.0752 | No |
| 4 | DTX2 | na | 7289 | 0.145 | 0.0878 | No |
| 5 | MAML3 | na | 7513 | 0.140 | 0.1136 | No |
| 6 | PTCRA | na | 7708 | 0.135 | 0.1391 | No |
| 7 | RBPJL | na | 9155 | 0.102 | 0.1209 | No |
| 8 | PSEN1 | na | 9208 | 0.101 | 0.1427 | No |
| 9 | CTBP2 | na | 10134 | 0.084 | 0.1355 | No |
| 10 | DTX3L | na | 10976 | 0.070 | 0.1275 | No |
| 11 | ADAM17 | na | 12204 | 0.053 | 0.1042 | No |
| 12 | DVL1 | na | 14350 | 0.026 | 0.0485 | No |
| 13 | HES1 | na | 14513 | 0.025 | 0.0495 | No |
| 14 | CREBBP | na | 14557 | 0.024 | 0.0538 | No |
| 15 | KAT2A | na | 14658 | 0.023 | 0.0562 | No |
| 16 | CIR1 | na | 14677 | 0.023 | 0.0609 | No |
| 17 | NOTCH2 | na | 14962 | 0.019 | 0.0572 | No |
| 18 | CTBP1 | na | 15097 | 0.018 | 0.0575 | No |
| 19 | DVL3 | na | 15800 | 0.011 | 0.0397 | No |
| 20 | NCOR2 | na | 15998 | 0.009 | 0.0360 | No |
| 21 | NOTCH1 | na | 16486 | 0.004 | 0.0229 | No |
| 22 | MAML1 | na | 16911 | -0.001 | 0.0108 | No |
| 23 | NUMB | na | 17077 | -0.002 | 0.0066 | No |
| 24 | NOTCH3 | na | 17507 | -0.006 | -0.0043 | No |
| 25 | RBPJ | na | 17904 | -0.010 | -0.0135 | No |
| 26 | NCSTN | na | 18220 | -0.013 | -0.0196 | No |
| 27 | HDAC1 | na | 18248 | -0.013 | -0.0174 | No |
| 28 | PSENEN | na | 19670 | -0.027 | -0.0521 | No |
| 29 | HES5 | na | 19916 | -0.029 | -0.0525 | No |
| 30 | NUMBL | na | 20516 | -0.035 | -0.0617 | No |
| 31 | HDAC2 | na | 20836 | -0.038 | -0.0621 | No |
| 32 | PSEN2 | na | 21046 | -0.040 | -0.0588 | No |
| 33 | SNW1 | na | 22228 | -0.053 | -0.0806 | No |
| 34 | LFNG | na | 22424 | -0.055 | -0.0735 | No |
| 35 | APH1A | na | 22976 | -0.062 | -0.0752 | No |
| 36 | DLL1 | na | 23464 | -0.068 | -0.0736 | No |
| 37 | EP300 | na | 23570 | -0.069 | -0.0606 | No |
| 38 | JAG1 | na | 23577 | -0.069 | -0.0448 | No |
| 39 | KAT2B | na | 23594 | -0.070 | -0.0292 | No |
| 40 | JAG2 | na | 23614 | -0.070 | -0.0136 | No |
| 41 | MAML2 | na | 24541 | -0.083 | -0.0211 | No |
| 42 | RFNG | na | 25582 | -0.100 | -0.0281 | No |
| 43 | DVL2 | na | 26784 | -0.122 | -0.0347 | No |
| 44 | DTX4 | na | 28490 | -0.162 | -0.0466 | No |
| 45 | NOTCH4 | na | 30385 | -0.219 | -0.0507 | No |
| 46 | DLL3 | na | 32115 | -0.298 | -0.0319 | No |
| 47 | DTX3 | na | 33908 | -0.465 | 0.0236 | No |
Table: GSEA details [plain text format]

  

Fig 2: KEGG\_NOTCH\_SIGNALING\_PATHWAY      
 Blue-Pink O' Gram in the Space of the Analyzed GeneSet

  

Fig 3: KEGG\_NOTCH\_SIGNALING\_PATHWAY: Random ES distribution      
 Gene set null distribution of ES for **KEGG\_NOTCH\_SIGNALING\_PATHWAY**
